# miR-200c suppression increases tau hyperphosphorylation by targeting 14-3-3γ in early stage of 5xFAD mouse model of Alzheimer's disease

**DOI:** 10.7150/ijbs.66604

**Published:** 2022-03-06

**Authors:** Hyunjun Park, Yeong-Bae Lee, Keun-A Chang

**Affiliations:** 1 Department of Health Sciences and Technology, Gachon Advanced Institute for Health Sciences and Technology (GAIHST), Gachon University, Incheon, 21999, Korea; 2 Neuroscience Research Institute, Gachon University, Incheon, 21565, Korea; 3 Department of Neurology, Gil Medical Center, College of Medicine, Gachon University, Incheon 21565, Korea; 4 Department of Pharmacology, College of Medicine, Gachon University, Incheon, 21999, Korea

**Keywords:** miR-200c, 14-3-3γ, GSK-3β, phosphorylated tau, Alzheimer's disease

## Abstract

**Background and Purpose:** Recently, several abnormally regulated microRNAs (miRNAs) have been identified in patients with Alzheimer's disease (AD). The purpose of this study was to identify abnormally expressed miRNAs and to investigate whether they affect pathological changes in AD in the 5xFAD AD mouse model.

**Experimental Approach:** Using microarray analysis and RT-qPCR, miRNA expression in the hippocampus of a 4-month-old 5xFAD mouse model of AD was investigated. A dual-luciferase assay was performed to determine whether the altered miR-200c regulates the translation of the target mRNA, Ywhag. Whether miR-200c modulates AD pathology was determined in primary hippocampal neurons and C57BL/6J mice transfected with miR-200c inhibitor. In addition, total miRNAs were extracted from the serums of 28 healthy age-matched controls and 22 individual participants with cognitive impairment, and RT-qPCR was performed.

**Key results:** miR-200c expression was reduced in the hippocampus of 5xFAD mice. In primary hippocampal neurons, miR-200c regulated the translation of 14-3-3γ and increased tau phosphorylation (p-tau) by increasing p-GSK-3β (GSK-3β phosphorylation). It was also confirmed that miR-200c inhibition in the hippocampus of C57BL/6J mice induces cognitive impairment and increases tau phosphorylation through 14-3-3γ activation. Finally, aberrant expression of miR-200c was confirmed in the blood serum of human AD patients.

**Conclusion and Implications:** Our results strongly suggest that dysregulation of miR-200c expression contributes to the pathogenesis of AD, including cognitive impairment through hyperphosphorylated tau.

## Introduction

Alzheimer's disease (AD) is the most representative neurodegenerative disease and is characterized by cognitive dysfunction and memory impairment. The neuropathological characteristics of AD include deposition of senile plaques due to the accumulation of amyloid β (Aβ), neurofibrillary tangles (NFT) through hyperphosphorylation of tau protein, and neuronal cell loss 1. NFT pathogenesis is less understood; however, it is known that NFT is derived from the abnormal aggregation of hyperphosphorylated microtubule-associated tau protein 2. Several other pathological mechanisms have been investigated as potential contributors to AD pathology. The density and distribution of neurofibrillary tangles in the brain correlate with the severity of dementia, with tau protein abnormally phosphorylated in the brain of patients with AD 2.

MicroRNAs (miRNAs) are small single-stranded non-coding RNAs that consist of 18-25 nucleotides and serve as post-transcriptional regulators of gene expression 3. The major function of miRNAs is to bind to the 3′ untranslated regions (3′ UTR) of the target mRNA, inhibiting the translation of the target mRNA or degrading the mRNA to downregulate gene expression 4, 5. Each mRNA with a 3′ UTR can be regulated by several miRNAs, and a single miRNA can bind to multiple mRNAs 6. Such as, miRNA abnormally regulates protein synthesis through mRNA expression regulation 7. Moreover, miRNA expression is specific to each cell, tissue, and disease 8.

Several miRNAs have been implicated in AD, which is associated with impairments in memory, behavior, and cognition 9. Recently, it has been reported that certain miRNAs appear to be dysregulated in AD, thereby leading to AD pathology 10-12. For example, miR-326 has been shown to decrease tau phosphorylation and neuronal apoptosis in AD 13. miR-200a-3p attenuates Aβ overproduction and tau hyperphosphorylation in AD pathology 14. miR-146a can affect AD pathological changes such as amyloid production, tau phosphorylation, cell death, synaptic formation, phagocytosis, and neuroinflammatory 15. However, it has not yet been confirmed whether any of the miRNAs that are dysregulated in AD are indeed associated with AD pathology.

In this study, we investigated the correlation between miRNA changes and pathological changes in 5xFAD mice. Through microarray analysis of miRNAs, we observed that the expression levels of several miRNAs, including miR-200c, were downregulated in the hippocampus of 4-month-old 5xFAD mice. The 14-3-3γ, which was explored as a target molecule of miR-200c, increased in the hippocampus of 4-month-old 5xFAD mice. In addition, 14-3-3γ, which was increased through miR-200c inhibition, increased GSK-3β activation and promoted tau phosphorylation. Also, miR-200c increased in serum of AD patients and confirmed association with MMSE. These results suggest that miR-200c can increase GSK-3β activity through 14-3-3γ regulation, thereby increasing the phosphorylation of tau.

## Material and Method

### Ethics statement

Animal studies are reported in compliance with the ARRIVE guidelines (Percie du Sert et al., 2020). All animal experiments were carried out in compliance with the Guide for Care and Use of Laboratory Animals of the National Institutes of Health and were approved by the Institutional Animal Care and Use Committee of the Lee Gil Ya Cancer and Diabetes Institute, Gachon University (LCDI-2018-0048).

The Institutional Review Board of the Gachon University Gil Medical Center approved the research protocol (GAIRB2013-264, GCIRB2016-015), and written signed consent, strictly abiding by the Declaration of Helsinki, was obtained from all the participants before participating via self-referral or referral from a family member.

### Animals

5xFAD (B6SJL) transgenic mouse (Jackson Laboratory, Bar Harbor, ME, USA) overexpress both mutant human APP 695, with the Swedish (K670N, M671L), London (V717I), Florida (I716V) familial Alzheimer's disease (FAD) mutations, and mutant human presenilin 1, harboring two FAD mutations (M146L and L286V). 5xFAD mice were maintained by crossing hemizygous transgenic mice with B6SJL F1/J mice (Jackson Laboratory). For miRNA inhibitor experiments, C57BL/6J mice were purchased from DBL (Eumseong, Korea). For behavioral tests, male mice were randomly assigned to groups to generate the same sample size consisting of Wild-type mice (WT, n=10), 5xFAD Tg mice (5xFAD, n=10), or negative control (Control, n=13); miR-200c inhibitor (Inhibitor, n=13). Animals were accommodated in an automatically controlled environment at 22 ± 2 °C and 50 ± 10 % relative humidity with ad libitum access to water and food under a 12-hour light and dark cycle. Food and water were provided freely while acclimating to the polycarbonate cage, with no more than five animals kept in one cage. It was bedding with sani-chips. If abnormal symptoms such as movement disability or sudden weight loss (weight loss of 20% or more compared to normal weight) are observed, euthanasia is decided by consulting a veterinarian or breeding officer. We put those mice in a sealed container and injected CO_2_ gas. After exposing it for more than 5 minutes, check for breathing and cardiac arrest.

### miRNA inhibitor transfection in C57BL/6J mouse brain

After mixing a miR-200c inhibitor (Qiagen, Hilden, Germany) with Invivofectamine 3.0 (Invitrogen), the mixture was incubated at 50 °C for 30 min. The mice were anesthetized using a mixture of Zoletil (12.5 mg/kg) and Rompun (17.5 mg/kg) and fixed on a stereotaxic injector; 0.5 nmol (1 nmol/µL) of the miR-200c inhibitor or negative control was injected into the hippocampus (A/P, -0.22 mm; ±M/L, 0.15 mm; D/V, -0.15 mm) at 0.5 μL/min using a Hamilton syringe. The Hamilton syringe was slowly removed, and the mice were sutured within 10 min. After surgery, the mice were stabilized for 3 days, following which behavioral tests were performed.

### Behavior tests

To confirm cognitive performance, we conducted two behavioral tests with WT and 5xFAD mice; the mice were allowed to rest for one day between each behavioral test. All the tests were performed by a technician who was blind to the genotype and group of the animals.

#### Passive avoidance test

The passive avoidance test was performed using an avoidance apparatus (Gemini Passive Avoidance System; San Diego Instruments, San Diego, CA, USA). The passive avoidance apparatus (42.5 cm wide and 35.5 cm long) consisted of a two-chamber box with a lighted and darkened compartment. The lighted box was illuminated by an LED house light (6 W) and was connected to the dark chamber, which was equipped with an electric grid floor. On the first day, the mice were placed in a lighted box and allowed to explore both compartments for 5 min. In the training step, the mice entered the dark box and an electric shock was applied at 0.3 mA for 3 s. The next day, the mice were placed in a lighted box, and the duration for which the mouse remained in the lighted box was recorded.

#### Morris water maze

The Morris water maze, with a diameter of 90 cm, was divided into four parts. The platform was placed on one of the four quadrants, and milk was added to the tank to cover the platform. The mice were trained to find the platform from each quadrant. We measured the time taken for the mice to reach the platform. This experiment was conducted four times a day for four days. A probe test was performed one day after training. In the probe test, the platform was removed, and the time spent in each quadrant was measured for determining whether the mice remembered the position of the platform. This experiment was performed by video recording using EthoVision XT 9 (Noldus, Wageningen, Netherlands).

### Tissue sampling

The mice were anesthetized with a mixture of Zoletil (12.5 mg/kg) and Rompun (17.5 mg/kg) and transcardially perfused with saline. Brain tissues were collected from both hemispheres; the tissues from one hemisphere were fixed with 4% PFA for preparing paraffin blocks for immunohistochemistry, and those from the other hemisphere tissues were dissected according to brain region and stored at -80 °C for molecular analysis.

### Microarray

RNA was extracted from the hippocampus of 4-month 5xFAD mice and WT mice using the miRNeasy Mini kit (Qiagen) according to the manufacturer's instructions. The extracted RNA samples were evaluated using a NanoDrop spectrophotometer (Thermo Fisher Scientific, Waltham, MA, USA). Total RNA quality was determined by RNA integrity number measurement using an Agilent 2100 Bioanalyzer. miRNA was labeled using Agilent's miRNA labeling agent and hybridization kit; subsequently, feature extraction was performed by detecting miRNA expressed using Agilent's DNA microarray scanner. The measured miRNA values were analyzed using GeneSpring, and the targets of the miRNAs were predicted using TargetScan, microRNA.org, and miRbase.

### Thioflavin S staining

Paraffin-embedded tissues were cut into 4 μm-thick coronal sections of the hippocampal region using a microtome (Thermo Fisher Scientific). The brain slides on a glass slide were placed in a 60 °C incubator for 1 h, rinsed with xylene for deparaffinization, and subjected to a series of washes with ethanol for dehydration. The brain slides were retrieved by treatment with 0.01 M citric acid (pH 6.0) for 10 min at 60 °C and washed with 0.5% Triton X-100 in PBS buffer.

The brain tissues were incubated with 0.1% Thioflavin S solution dissolved in 30% ethanol for 10 min. The brain tissues were washed with phosphate-buffered saline and covered with a coverslip, along with a mounting solution. The brain was imaged using a Nikon DS-Qi2 camera (Nikon Corporation, Tokyo, Japan) and a Nikon Eclipse Ts2 microscope (Nikon Corporation).

### RT-qPCR

Total RNA or the specific miRNA fraction was extracted using the miRNeasy Mini kit (Qiagen), and fifty nanogram of RNA was processed for cDNA synthesis using Taqman MicroRNA assay (TOYOBO, Osaka, Japan) or SYBR Green real-time PCR master mix (TOYOBO) according to the manufacturer's instructions. To quantify the miRNA expression levels, SYBR Green miRNA assay-based RT-qPCR was performed on a real-time PCR system (Applied Biosystems, Waltham, MA, USA) using the ΔΔCt method. All data were normalized to snoRNA 202 (Applied Biosystems) in the animals and to U6 snRNA (Applied Biosystems) or GAPDH in primary hippocampal neurons. Table 1 lists the sequences of the primers.

### Western blot

Proteins were extracted in RIPA buffer (50 mM Tris pH 8.0, 150 mM NaCl, 1% NP-40, and 0.1% SDS), and protein concentrations were measured using a Bradford protein assay kit (Bio-Rad, Hercules, CA, USA). The protein extracts were denatured at 95 °C for 10 min and separated using SDS-PAGE. The proteins were transferred to a polyvinylidene difluoride (PVDF) membrane (Merck Millipore, Burlington, MA, USA). The membrane was incubated for 1 h in 3% BSA in TBS-T at room temperature and incubated overnight with primary antibodies such as 14-3-3γ, AT-8, AT-180, Tau H-150, β-actin, or GADPH (Table 2). After the membranes were washed three times in Tris-buffered saline with Tween 20 (TBS-T), they were incubated with goat anti-rabbit or goat anti-mouse IgG conjugated with horseradish peroxidase (HRP) in 3% BSA and visualized using the D-plus ECL pico system (ELPIS-biotech, Daejeon, Korea) or Immobilon Western chemiluminescent HRP substrate (Merck Millipore, Burlington, MA, USA) for visualizing protein bands. Signals were detected using blue-sensitive medical X-ray films (Agfa, Mortsel, Belgium) or iBright™ FL1500 Imaging System (Invitrogen, Carlsbad, CA, USA) and the intensity was analyzed using the Image J software (v1.4.3.67, NIH, USA).

### Cell culture

Pregnant Sprague-Dawley (SD) rats were purchased from Koatech (Pyeongtaek, Korea). Hippocampal tissues were dissected from embryonic day 17 (E17) SD rat embryos and dissociated with 0.25% trypsin solution (Invitrogen, Carlsbad, CA, USA). The isolated cells were plated on poly-ʟ-lysine-coated plates (Sigma-Aldrich, St. Louis, MO, USA) at a density of 5 × 10^4^ cells/well. Primary hippocampal neurons were cultured in Neurobasal media (Invitrogen) supplemented with 2 mM L-glutamine (Sigma-Aldrich), B27 supplement (Invitrogen), and 1× antibiotic Pen/Strep (Invitrogen) and were incubated at 37 °C in Dulbecco's modified Eagle's medium (DMEM; Welgene, Gyeongsan, Korea) under 5% circulating CO_2_ for 10 days. The culture medium was changed every 2-3 days. The primary hippocampal neuron culture protocol was approved by the Institutional Animal Care and Use Committee of the Lee Gil Ya Cancer and Diabetes Institute, Gachon University (LCDI-2018-0137).

HEK293 cells and SH-SY5Y cells were cultured in DMEM supplemented with 10% heat-inactivated fetal bovine serum (FBS, Hyclone Laboratories Inc, Logan, UT, USA) in a humidified atmosphere of 5% CO_2_ at 37 °C and passaged for 2-3 days. The AR-A014418 (Sigma-Aldrich, St. Louis, MO, USA) and LiCl (Sigma-Aldrich, St. Louis, MO, USA), GSK-3 inhibitors, were treated with SH-SY5Y cell, and protein samples were extracted 4 hours later.

### Luciferase activity assay

Luciferase reporter assays were performed using HEK 293 cells. A vector containing the full-length 3′ UTR of Ywhag was purchased from Addgene (Watertown, MA, USA). Briefly, HEK 293 cells were transfected with psiCHECK-2-Ywhag-3′ UTR vector and miR-200c using Lipofectamine 2000 (Thermo Fisher Scientific). Luciferase activity was determined 72 h after transfection, and the reporter assay was performed according to the manufacturer's protocol (Dual-Glo Luciferase Assay System, Promega, Madison, WI, USA). Firefly luciferase activity (mean ± SEM) was normalized to Renilla luciferase and expressed as a percentage of the negative control.

### miRNA transfection

Lipofectamine 2000 (Invitrogen) with the synthetic miR-200c mimic, inhibitor, or negative control (control) (Qiagen) was added to the Opti-MEM medium (Invitrogen) and incubated for 5 min. Both solutions were mixed and SH-SY5Y cell or primary hippocampal neurons were incubated with this mixture for 10 min at room temperature. After 2 or 7 days of incubation, RNA or protein was extracted from the SH-SY5Y cell or primary hippocampal neurons transfected with the miR-200c mimic, inhibitor, or control.

### Participants, serum separation and miRNA extraction

A total of 50 subjects aged 61-89 years were recruited from Gachon University Gil Medical Center, Incheon, Korea, including 28 healthy age-matched control subjects and 22 cognitive impairments confirmed according to the criteria described in our previous report 16. Among individuals with subjective cognitive dissatisfaction, the cognitive impairment was examined using the Mini-Mental State Examination (MMSE) and > 26 of 30 point were excluded in AD patients group 17. Subjects scoring below 26 were subjected to detailed neuropsychological testing, including the Clinical Dementia Rating (CDR; scores > 0.5) and Global Deterioration Scale (GDS; scores > 3), which are broadly accepted measures for dementia. Patients with comorbidities were excluded. Patients were diagnosed according to American Psychiatric Association DSM-IV criteria. All clinical tests were performed by investigators blinded to the subjects' genetic status; however, the blinded condition could not realistically be maintained for overtly demented subjects. Table 3 summarizes the clinical characteristics of the study group.

Ten milliliters of whole blood from an individual participant were collected in a heparin-based anticoagulant tube. The blood was kept at RT for 30 min and centrifuged for 10 min at 800 g. The supernatant (Serum) was collected, and a protease inhibitor cocktail (535140; EMD Biosciences, Inc., Darmstadt, Germany) and phosphatase inhibitor cocktail (P5726 and P0044; Sigma-Aldrich, Inc., St. Louis, Missouri, USA) were added. The serum was frozen at -80°C and then thawed immediately when used in the experiment. Total miRNA was extracted from serum according to the instructions of the miRNeasy Serum/Plasma kit (Qiagen), and the concentration and purity of RNA were determined by ultraviolet spectrophotometry.

### Data and statistical analysis

All statistical analyses were performed using the GraphPad Prism 8.4.2 (679) software (GraphPad Software Inc., San Diego, CA, USA), and outliers were removed using the Outlier calculator (significance level: Alpha = 0.05) in the GraphPad Prism software. Statistical analysis was undertaken only for experiments where the sample size was at least *n* = 5 per group. The group size is the number of independent values and the statistical analysis was done using these independent values. All data are presented as the mean ± standard error. Differences in the collected data between groups were analyzed using the student's t-test or one-way ANOVA test. P-values less than 0.05 were considered statistically significant (*p<0.05; **p<0.01; ***p<0.001; and ****p<0.0001).

## Result

### 5xFAD mice show memory impairment and AD pathology

**Figure 1:**
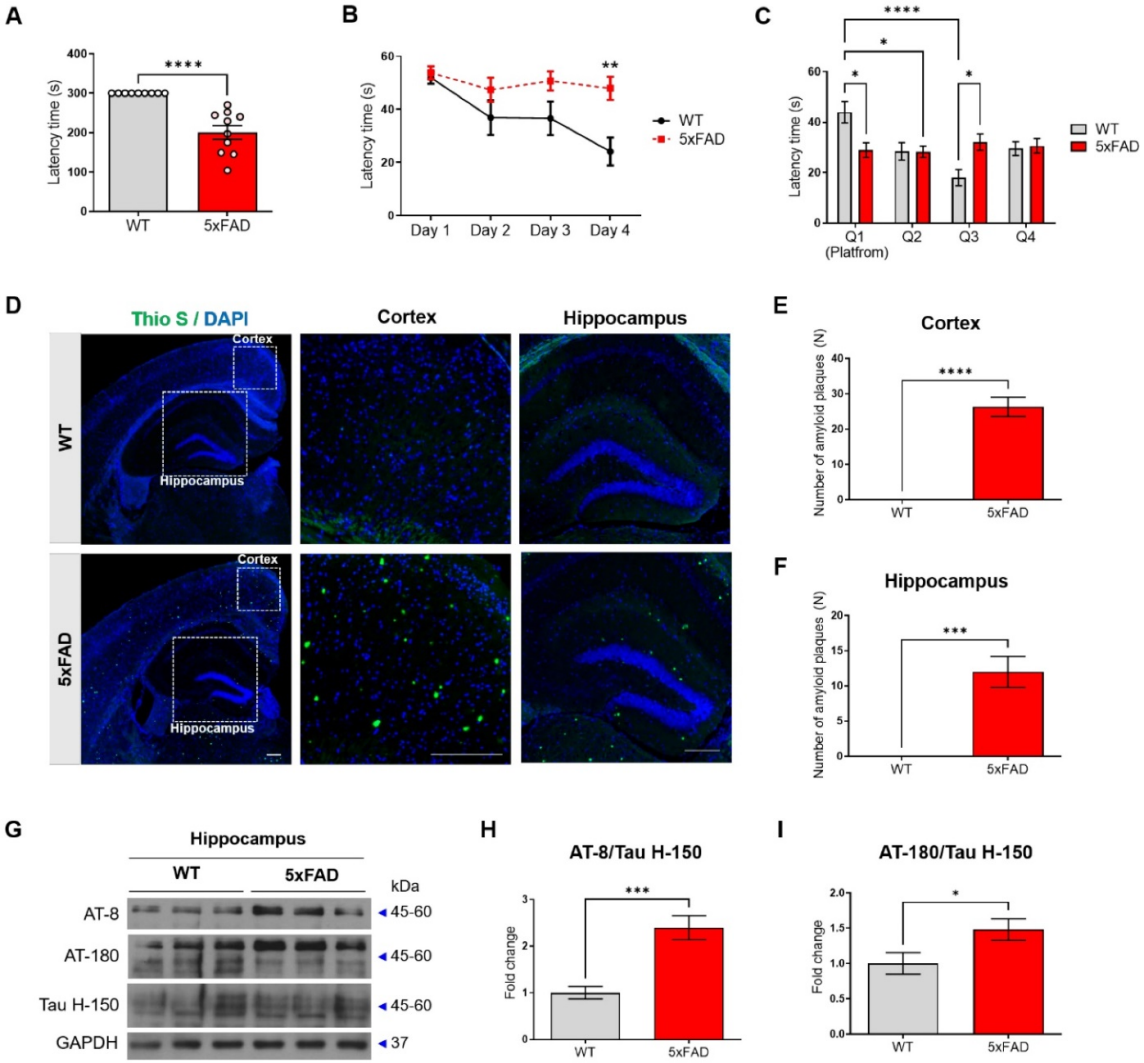
** Cognitive impairment and AD pathology appear in 4-month-old 5xFAD mice.** (A) The latency time to the dark chamber was measured in the passive avoidance test. (B) The escape latency time in the Morris water maze (MWM) test was measured for 4 days. (C) After one day of rest after the MWM final training, the latency time for each quadrant was measured in the probe test. (D-F) The number of amyloid β plaques was analyzed by Thioflavin S (Thio S) staining and counterstained with DAPI. (D) Representative slices were shown for WT and 5xFAD mice brain (40× magnification), hippocampus (100× magnification), and cortex (200× magnification) (All scale bar = 200μm). (E-F) Quantified results of Thio S in the hippocampus (E) and the cortex (F). (G-I) Expression of phosphorylated tau proteins (AT-8 and AT-180) and tau protein (Tau H-150) in the hippocampus was analyzed using Western blotting analysis. The immunoreactivities of AT-8/Tau H-150 (H) and AT-180/Tau H-150 (I) were relatively quantified by Image J and normalized to the GAPDH internal control. Data are shown as the mean ± SEM (*n =* 10 mice per group). Data from the Morris water maze test and Probe test were analyzed by two-way ANOVA test, and other data were analyzed by student's T-test. ***p<0.001 versus WT mice.

First, we confirmed that the pathological features of AD, memory impairment, Aβ plaques, and hyperphosphorylated tau, appear in the 4-month-old 5xFAD mice. To evaluate the cognitive impairment of the 5xFAD mice, we performed a passive avoidance test and the Morris water maze test. In the passive avoidance test, two groups of mice aged 4 months were evaluated for short-term memory; the latency times were significantly reduced in the 5xFAD mice compared to that in the WT mice (Figure 1A). In the Morris water maze test, the escape time to the platform was measured during the training period. According to the results of the Morris water maze test, which was performed for identifying hippocampus-dependent spatial and working memory, the latency times in the 5xFAD mice were significantly reduced compared to that in the WT mice (Figure 1B).

In the probe test, the 5xFAD mice displayed a decrease in the time spent in the platform zone compared with the WT mice (Figure 1C). There was no significant difference between the time spent by the 5xFAD mice in Q1 and that spent in the other quadrants (Figure 1C). To confirm the pathological characterization of the 5xFAD mice, Aβ deposition was confirmed using Thioflavin S staining, and the level of hyper-phosphorylated tau was confirmed using western blot analysis with AT-8 and AT-180 antibodies. The 5xFAD mice exhibited a higher amount of Aβ plaques in both the hippocampus and cortex compared with the WT mice (Figure 1D-F). In addition, the 5xFAD mice exhibited an increase in the level of phosphorylated tau compared with the WT mice (Figure 1G-I). These data confirmed that the 4-month-old 5xFAD mice showed cognitive impairment, increased Aβ plaques, and increased levels of phosphorylated tau.

### miR-200c is downregulated in the hippocampus of 4-month-old 5xFAD mice

**Figure 2:**
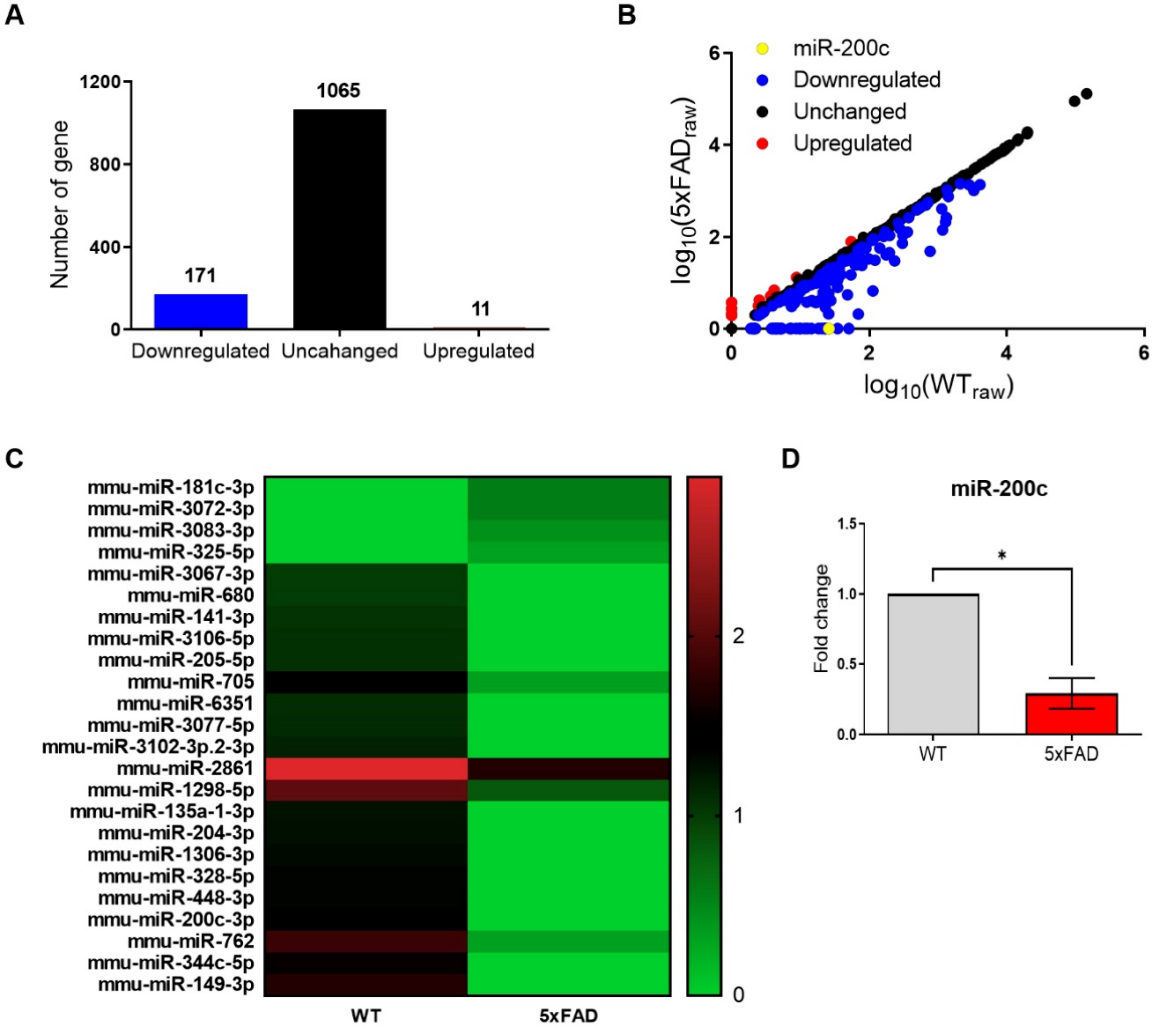
** Aberrant expression of miR-200c in the hippocampus of 4-month-old 5xFAD mice brains.** (A) Expression of miRNA in the hippocampus of 4-month-old 5xFAD mice brain using microarray analysis (*n* = 3 mice per group). (B) Altered levels of miRNAs in 5xFAD mice relative to WT mice. Data are shown as the number of changed miRNA. (C) The miRNAs profiles differentiate the 5xFAD group from the WT group. Hierarchical clustering of 24 miRNAs whose expression was significantly altered in the 5xFAD and WT groups. The color stands for the intensity of the signal. (D) Reduced expression of miR-200c in the hippocampus of 4-month-old 5xFAD mice brains compared with WT mice was confirmed using RT-qPCR (*n =* 6 mice per group). Data represent the mean ± SEM. Data were analyzed by student's T-test. *p<0.05 versus WT.

On the basis of these data, we performed a microarray analysis to identify dysregulated miRNA expression in the hippocampus of the 4-month-old 5xFAD mice compared with that in age-matched WT mice (Figure 2). Among the several miRNAs with altered expression, 11 miRNAs were upregulated and 171 miRNAs were downregulated (Figure 2A, B). miR-200c was the most significantly decreased miRNA in the hippocampus of the 4-month-old 5xFAD mice, relative to the corresponding level in the WT mice (Figure 2B, C). The expression profiles of 24 miRNAs differentially regulated between the 5xFAD and WT groups were used to separate samples into biologically interpretable groups (Figure 2C). Among these, 4 miRNAs were upregulated by more than 1.5-fold in the 5xFAD group compared with the WT group, while 20 miRNAs were downregulated by more than 10-fold. To confirm the miR-200c expression levels, we performed RT-qPCR, which revealed that the miR-200c expression was significantly reduced in the hippocampus of the 5xFAD mice (Figure 2D). These data indicated that miR-200c downregulated in hippocampus of the 4-month-old 5xFAD mice.

### miR-200c regulates the post-translation of 14-3-3γ by directly binding to the 3′ UTR of 14-3-3γ mRNA

**Figure 3:**
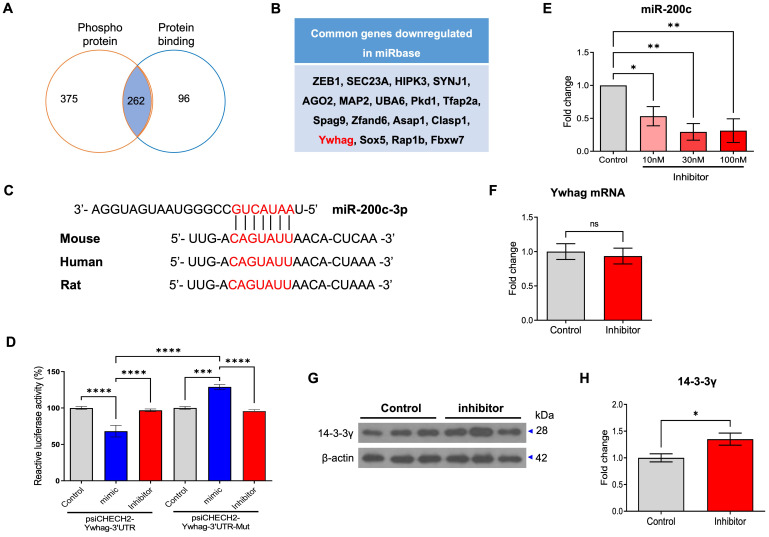
** miR-200c directly target the 3'UTR of Ywhag mRNA.** (A) The genes belonging to phospho-protein and protein binding were shown to be Venn diagram by category analysis in DAVID. (B) List of common genes downregulated by miR-200c in the miRbase database. (C) Conservation analysis of predicted miR-200c binding sites in the Ywhag 3'-UTR mRNA across different species. (D) The relative luciferase activity was measured 72 hr after transient transfection with psiCHECK2-Ywhag 3'UTR plasmid or mutant form of psiCHECK2-Ywhag 3'UTR (psiCHECK2-Ywhag 3'UTR-Mut) plasmid with miR-200c mimic, inhibitor, or control into HEK293 cells. (E) Changes in expression of miR-200c were evaluated in primary hippocampal neurons transfected with miR-200c inhibitors (0, 10, 30, 100nM). (F) The expression of Ywhag mRNA in primary hippocampal neurons was evaluated using RT-qPCR (*n =* 6 per group). (G-H) The expression of 14-3-3γ protein was analyzed in miR-200c inhibitor-transfected primary hippocampal neurons using Western blotting analysis. The immunoreactivity of 14-3-3γ was relatively quantified by Image J and normalized to the β-actin internal control (*n* = 6-12 per group). Data represent the mean ± SEM. Data were replicated in a minimum of independent twice cultured samples. Data from the Luciferase activity were analyzed by two-way ANOVA test, data of miR-200c expression were analyzed by one-way ANOVA test, and other data were analyzed by student's T-test. *p<0.05, **p<0.01, ***p<0.001, ****p<0.0001, and ns = non-significant.

Next, we identified mRNA that can be regulated by miR-200c using TargetScan 7.1 and analyzed the functional annotation using DAVID (Table 3). These annotations identified the top five categories, among them, we selected mRNAs that can regulate two or more functions and are related with AD pathogenesis. Phosphoproteins and protein binding were identified as being involved in AD mechanisms among five categories, 262 mRNA was identified in both two categories (Figure 3A). Among them, 17 genes were indexed with the top 5% miR-200c target gene in the miRBase, and Ywhag was included as a gene related to AD (Figure 3B). Ywhag is a gene belonging to the 14-3-3 protein family and encodes 14-3-3 protein γ 18. In addition, using TargetScan 7.1, we investigated the potential of miR-200c to bind to and modulate the Ywhag 3'-UTR mRNA in human, rat and mouse (Figure 3C). miR-200c binds to the 3′ UTR region of 14-3-3γ, which is expected to regulate translation. Using luciferase analysis, we confirmed whether 14-3-3γ could be a target of miR-200c. Compared with control cells, HEK293 cells co-transfected with a psiCHECK-2-Ywhag-3′ UTR plasmid and a miR-200c mimic indicated that the expression of miR-200c significantly suppressed the luciferase activity (Figure 3D).

To further determine whether the translation of 14-3-3γ is regulated by miR-200c, we transfected neuronal cells (SH-SY5Y and primary hippocampal neurons) with the mimic or inhibitor of miR-200c and then examined changes in the RNA and protein levels of 14-3-3γ. To evaluate the modulation of the 14-3-3 translation by miR-200c, we transfected the mimic or inhibitor of miR-200c in a dose-dependent manner and transfection efficiency was assessed in SH-SY5Y neuronal cells using RT-qPCR (Supplementary Figure 1A, B). The miR-200c mimic did not change the expression of the endogenous 14-3-3γ protein (Supplementary Figure 1C, D). However, the miR-200c inhibitor significantly increased the expression of the endogenous 14-3-3γ protein (Supplementary Figure 1E, F). Transfection efficiency was assessed in primary hippocampal neurons using RT-qPCR, and a concentration of 30 nM was chosen as the optimal inhibitor dose (Figure 3E). The miR-200c inhibitor did not affect the level of endogenous 14-3-3γ mRNA expression (Figure 3F). However, the miR-200c inhibitor significantly increased the expression of endogenous 14-3-3γ protein (Figure 3G, H). These results suggest that miR-200c post-translationally regulates 14-3-3γ by binding to the 3′ UTR of 14-3-3γ mRNA rather than by mRNA cleavage.

### miR-200c inhibition enhances p-GSK-3β and p-tau through 14-3-3γ regulation

**Figure 4:**
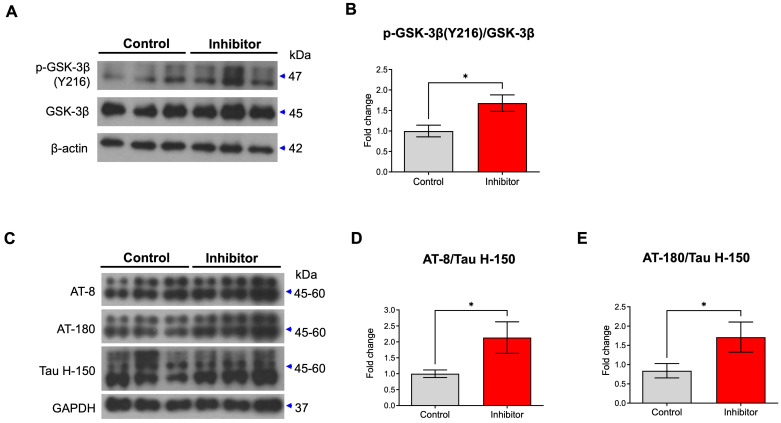
** Changes in expressions of p-GSK-3β and p-tau by miR-200c inhibition in primary hippocampal neurons.** (A) Expression of phosphorylation of GSK-3β (Y216) and GSK-3β protein in primary hippocampal neurons transfected with miR-200c inhibitor were analyzed with Western blotting. (B) The immunoreactivity of p-GSK-3β/GSK-3β was relatively quantified by Image J and normalized to the β-actin internal control. (C) Expression of phosphorylated tau proteins (AT-8 and AT-180) and tau protein (Tau H-150) in primary hippocampal neurons transfected with miR-200c inhibitor were analyzed with Western blotting. (D-E) The immunoreactivities of AT-8/Tau H-150 (C) and AT-180/Tau H-150 (E) were relatively quantified by image J and normalized to the GAPDH internal control. Data are shown as the mean ± SEM (*n =* 6-12 per group). Data were replicated in a minimum of independent twice cultured samples. Data were analyzed by student's T-test. *p<0.05 versus control.

Previously, 14-3-3 was reported to enable glycogen synthase kinase-3 β (GSK-3β) to bind to tau by forming a brain microtubule-associated tau phosphorylation complex 19. 14-3-3 binds to and mediates the phosphorylation of the microtubule-associated tau protein through GSK-3β in the brain 20. In addition, p-GSK-3β phosphorylates multiple sites of tau 21. Thus, inhibition of miR-200c could enhance the increases in GSK-3β and tau phosphorylation by increasing the translation of the 14-3-3γ protein. We transfected the mimic or inhibitor of miR-200c in primary hippocampal neurons or SH-SY5Y and assessed the levels of p-GSK-3β and p-tau activation (Supplementary Figure 2, Figure 4). The miR-200c mimic doesn't affect the level of GSK-3β phosphorylation in SH-SY5Y cells (Supplementary Figure 2A, B). On the other hand, miR-200c inhibitor increased the level of GSK-3β phosphorylation (Y216) in SH-SY5Y cells (Supplementary Figure 2C, D). The miR-200c mimic doesn't affect the level of tau phosphorylation using AT-8 and AT-180 antibodies (Supplementary Figure 2E-G). However, the group transfected with the miR-200c inhibitor showed significantly higher levels of tau phosphorylation than the control group through western blot analysis using AT-8 and AT-180 antibodies (Supplementary Figure 2H-J). Similarly, we transfected the inhibitor of miR-200c in primary hippocampal neurons. Treatment of miR-200c inhibitor increased GSK-3β phosphorylation at Y216 (Figure 4A, B). In addition, transfected with the miR-200c inhibitor showed significantly higher levels of tau phosphorylation than the control group through western blot analysis using AT-8 and AT-180 antibodies (Figure 4C-E).

**Figure 5:**
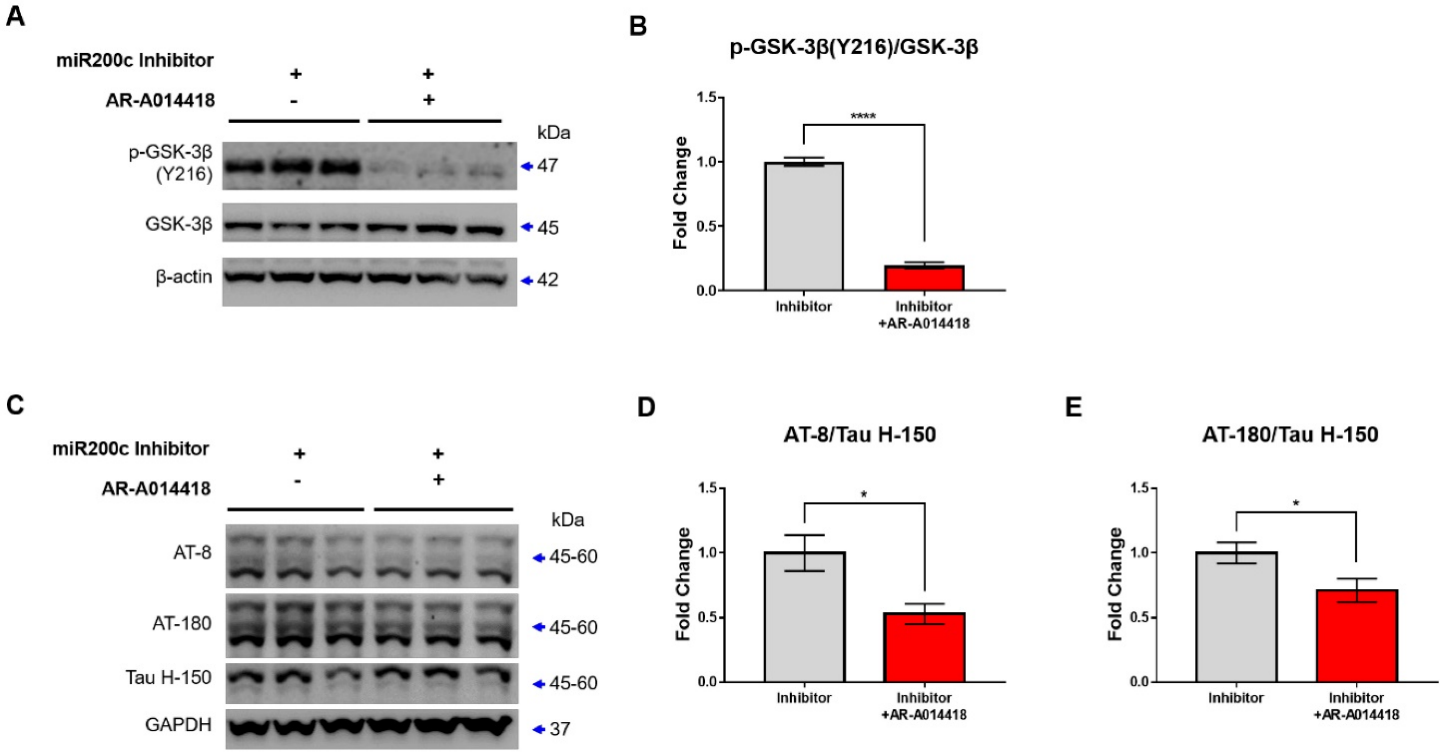
**GSK-3β inhibitor reduces the Tau phosphorylation.** (A) The expression of p-GSK-3β (Y216), GSK-3β protein in SH-SY5Y after treatment of AR-A014418 (GSK-3β inhibitor) were analyzed with Western blotting. (B) The immunoreactivity of p-GSK-3β/GSK-3β were relatively quantified by Image J and normalized to the β-actin internal control. (C) Expression of phosphorylated tau proteins (AT-8 and AT-180) and tau protein (Tau H-150) in SH-SY5Y after treatment of AR-A014418 were analyzed with Western blotting analysis. (D-E) The immunoreactivities of AT-8/Tau H-150 (D), and AT-180/Tau H-150 (E) were relatively quantified by Image J and normalized to the GAPDH internal control. Data represent the mean ± SEM. Data were replicated in a minimum of independent three times cultured samples. All data were analyzed by one-way ANOVA test. *p<0.05, **p <0.01, ***p <0.001, ****p<0.0001, and ns = non-significant.

To determine whether p-GSK-3β (Y216), which was increased by miR-200c inhibitor, increased leading to tau phosphorylation, GSK-3β phosphorylation or tau phosphorylation levels were determined after treatment with GSK-3β inhibitor (AR-A014418 or LiCl). GSK-3β inhibitor, AR-A014418, reduced p-GSK-3β (Y216) levels increased by miR-200c inhibitor, but LiCl did not change (Supplementary Figure 3). In previous studies, AR-A014418 decreased p-GSK-3 (Y216) and decreased p-tau in SH-SY5Y cells, but LiCl was not 22. This means that AR-A014418 is more suitable for inhibiting tau phosphorylation by controlling GSK-3 phosphorylation than LiCl. To determine whether the increased tau phosphorylation by miR-200c inhibitor is regulated by GSK-3β phosphorylation, the level of phosphorylated tau was checked after treatment with GSK-3 inhibitor, AR-A014418 (Figure 5). GSK-3β inhibitor decreased p-GSK-3β (Y216) levels in the miR-200c inhibitor + AR-A014418 group compared to the miR-200c inhibitor group (Figure 5A, B). In addition, phosphorylated tau was decreased in the miR-200c inhibitor + AR-A014418 group compared to the miR-200c inhibitor group (Figure 5C-E). This suggests that tau phosphorylation increased by miR-200c inhibition was due to increased GSK-3β phosphorylation. This suggests that the decrease in miR-200c increases the level of 14-3-3γ, which may increase tau phosphorylation through the activation of GSK-3β.

### 14-3-3 γ and p-GSK-3β is increased in hippocampus of 4-month-old 5xFAD mice

**Figure 6:**
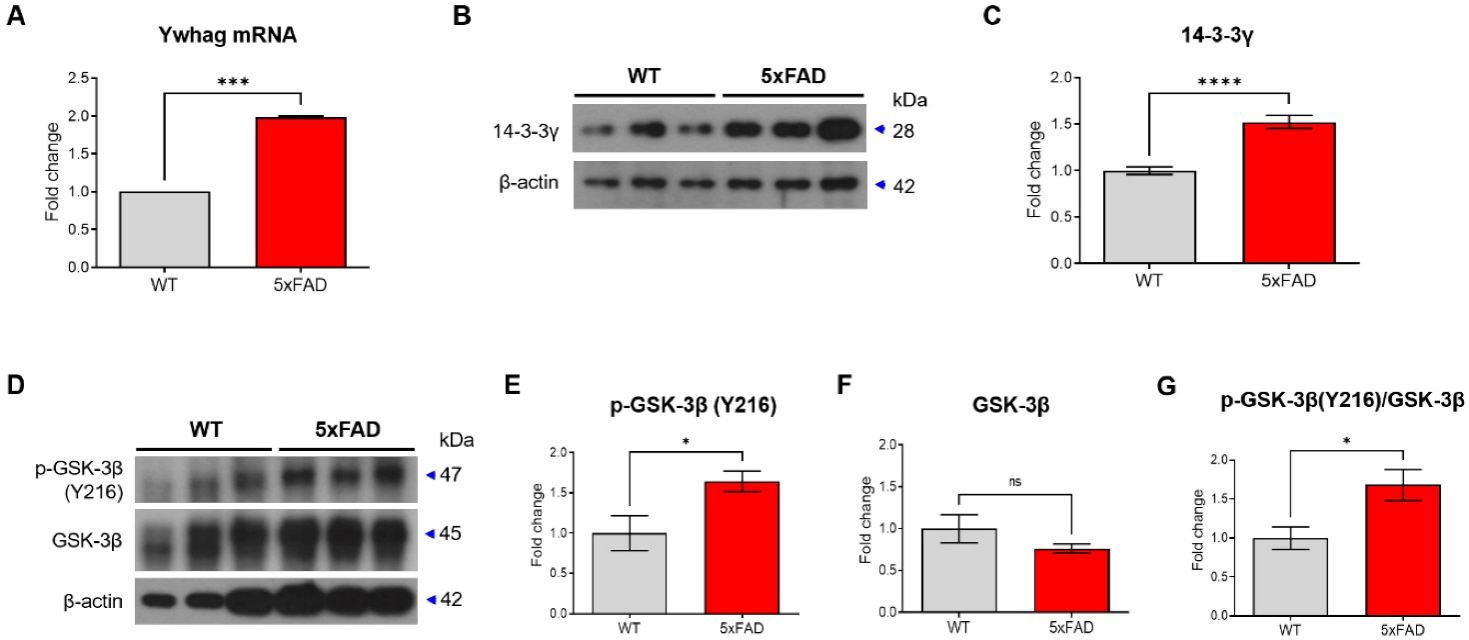
** Ywhag as the target of miR-200c in the hippocampus of 4-month-old 5xFAD mice.** (A) Expression of Ywhag in the hippocampus of 4-month-old 5xFAD mice brain was analyzed using RT-qPCR. (B) Expression of 14-3-3γ protein in hippocampus were analyzed with Western blotting analysis. (C) The immunoreactivity of 14-3-3γ was relatively quantified by Image J and normalized to the β-actin internal control. (D) Western blot was performed to measure the activity of GSK-3β in 4-month-old 5xFAD mice. (E-G) The immunoreactivity of phosphorylation of GSK-3β (Y216) and GSK-3β protein were relatively quantified by Image J and normalized to the β-actin internal control. Data are shown as the mean ± SEM (*n =* 6 per group). Data were analyzed by student's T-test. *p<0.05, and ****p<0.0001 versus WT.

We examined changes in the levels of 14-3-3γ and p-GSK-3β due to a decrease in miR-200c in hippocampus of 4-month-old 5xFAD mice. We observed that the level of Ywhag mRNA was increased in the hippocampus of the 4-month-old 5xFAD mice compared with that in the WT mice (Figure 6A). In addition, 14-3-3γ protein expression was increased in the hippocampus of the 4-month-old 5xFAD mice (Figure 6B, C). Additionally, p-GSK-3β (Y216) expression in the 5xFAD mice was increased compared with that in the WT mice (Figure 6D-G). These data indicated an increase in the 14-3-3γ and p-GSK-3β levels in the hippocampus of the 4-month-old 5xFAD mice.

### Intra-hippocampal injection of miR-200c inhibitor induced memory impairment and increased tau phosphorylation in C57BL/6J mice

**Figure 7:**
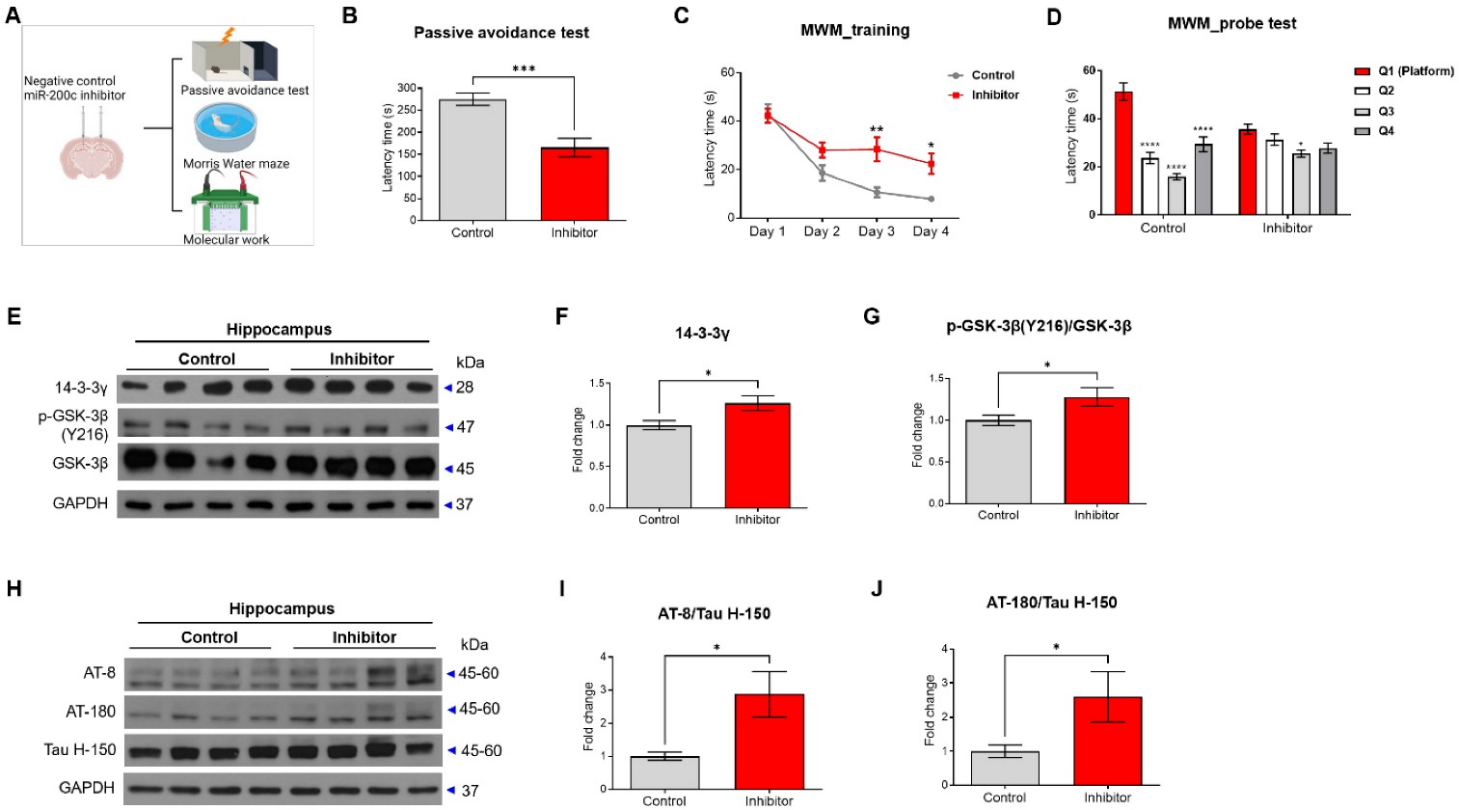
** Cognitive impairment in C57BL/6J mice transfected with miR-200c inhibitor.** (A) Scheme of miR-200c inhibitor injection into the hippocampus of C57BL/6J mice and subsequent experiments. C57BL/6J mice were transfected with miR-200c inhibitor or negative control before the behavior test (*n* = 13 mice per group). (B) The latency time to the dark chamber was measured in the passive avoidance test. (C) The escape latency time in the Morris water maze (MWM) test was measured for 4 days. (D) After one day of rest after the MWM final training, the latency time for each quadrant was measured in the probe test. (E) Expression of 14-3-3γ and phosphorylation of GSK-3β proteins (Y216) and GSK-3β protein in the hippocampus was analyzed with Western blotting. (F-G) The immunoreactivities of 14-3-3γ (F) and p-GSK-3β/GSK-3β (G) were relatively quantified by Image J and normalized to the GAPDH internal control. (H) Expression of phosphorylated tau proteins (AT-8 and AT-180) and tau protein (Tau H-150) in the hippocampus were analyzed using Western blotting analysis. (I-J) The immunoreactivities of AT-8/Tau H-150 (I) and AT-180/Tau H-150 (J) were relatively quantified by Image J and normalized to the β-actin internal control (*n* = 8 mice per group). Data are shown as the mean ± SEM. Data from the probe test of Morris water maze was analyzed by two-way ANOVA test, and other data were analyzed by student's T-test. *p<0.05, **p<0.01, ****p<0.0001 versus Control.

To determine whether reducing miR-200c increases the phosphorylation of tau protein, the miR-200c inhibitor or control was injected into the hippocampus of C57BL/6J mice, and the memory was tested using the passive avoidance test and the Morris water maze test (Figure 7A). In the passive avoidance test, the mice treated with the miR-200c inhibitor showed reduced latency times compared to the control mice (Figure 7B). In the Morris water maze test, the mice treated with the miR-200c inhibitor showed increased escape latency times compared to the control mice (Figure 7C, D).

The related 14-3-3γ-p-GSK-3β-p-tau pathway was evaluated in the hippocampus of the mice (Figure 7E-G). The 14-3-3γ protein level was increased in the mice treated with the miR-200c inhibitor compared to that in control mice (Figure 7F). Similarly, the p-GSK-3β protein level was increased in the C57BL/6J mice treated with the miR-200c inhibitor compared to that in the control mice (Figure 7G). Additionally, injection of the miR-200c inhibitor into the hippocampus of the C57BL/6J mice increased phosphorylated tau levels compared to control mice, as determined by western blot analysis using AT-8 and AT-180 antibodies (Figure 7H-J).

### miR-200c is down-regulated in the human AD serum

Next, we performed RT-qPCR in human blood serum to determine whether miR-200c expression was regulated in human AD. Table 4 summarizes the clinical and demographic characteristics of the study population. We divided subjects into an age-matched control group (control, *n* = 28) and AD group (*n* = 22) according to neurocognitive test scores as indicated by the MMSE, CDR, GDS scores. We compared the average age of the group to exclude the effects of age, one of risk factor in AD, and it showed similar distribution among the groups (Control, 69.89 ± 1.10; AD, 76.45 ± 1.89 years).

**Figure 8:**
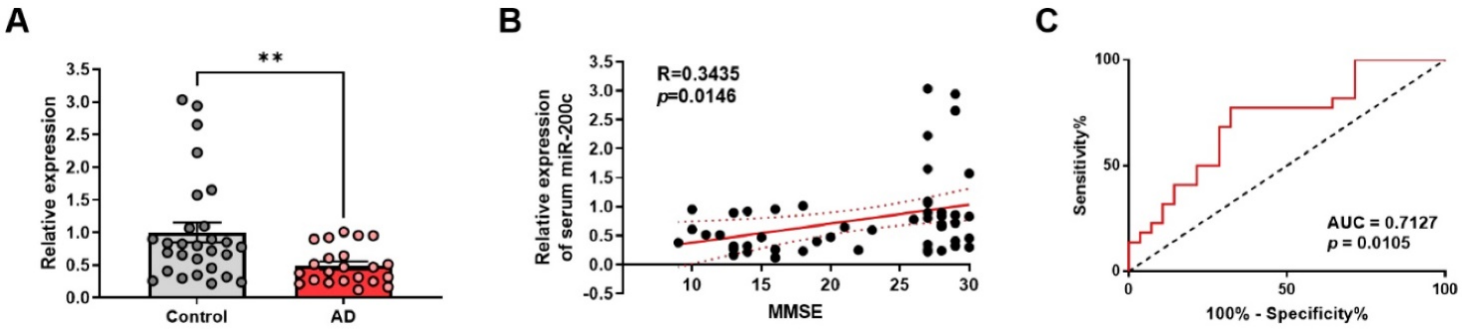
** Aberrant expression of miR-200c in the blood serum of human AD patients.** (A) Relative expression of miR-200c in the human blood serum using RT-qPCR. Data are shown as the mean ± SEM (Control, *n* = 28; AD, *n* = 22). Data were analyzed by student's T-test. *p<0.05, versus control. (B) The correlation between relative expression of serum miR-200c and MMSE scores was assessed by the nonparametric Spearman's rank correlation test. Graphs show regression lines with 95% confidence intervals. Serum miR-200c levels were significantly correlated with MMSE scores. (C) Receiver operating characteristic (ROC) analyses of serum miR-200c. AUC, area under the curve.

As shown in Figure 8A, relative expression of miR-200c in human blood serum were significantly down-regulated in AD compared to control group (Control, 1.00 ± 0.15; AD, 0.4943 ± 0.06; *p* = 0.0071). To further assess the relationship between miR-200c and AD, a correlation analysis between miR-200c levels and MMSE scores was performed. The results showed that serum miR-200c levels were positively correlated with MMSE scores (r = 0.3435, *p* = 0.0146, Figure 8B). To evaluate the diagnostic efficacy of miR-200c in AD, the ROC curve of miR-200c was drawn (AUC = 0.7127, *p* = 0.0105, Figure 8C). These results indicate that serum miR-200c levels can serve as reliable biomarkers for AD, further suggesting that miR-200c detection in the blood can be applied to the diagnosis of AD.

## Discussion

In AD, multiple genes and global brain regions are affected, and tau phosphorylation increases as the disease progress 23. Increased hyperphosphorylated tau affects memory impairment and cytotoxic damage to neuronal loss 24, 25. This suggests that AD may be caused by memory impairment and AD pathology due to the dysregulation of multiple genes. miRNAs are involved in the pathology of various diseases such as AD 26-28. In this study, we investigated whether dysregulated miRNAs are associated with AD pathology. Therefore, we identified abnormally expressed miRNAs by performing a microarray analysis in 4-month-old 5xFAD mice that showed memory impairment, Aβ plaques, and hyper-phosphorylated tau. The microarray results showed that 11 miRNAs were downregulated and 171 miRNAs were downregulated, of which miR-200c was significantly decreased.

miR-200c is a member of the miR-200 family, which consists of miR-200a, miR-200b, miR-429, and miR141, and is located on chromosome 12p13.31 in humans. miR-200c has been reported to play a role in cancer metastasis, cell proliferation, and cell apoptosis, and its association with AD remains controversial 29-31. In a previous study, miR-200a was downregulated in 9-month-old APP/PS1 mice 14, whereas another study reported that miR-200a was increased in the hippocampus of APPswe/PSΔE9 mice. In addition, miR-200b/c was reported to be increased in the cortex of the Tg2576 mouse brain, which is associated with Aβ peptide-induced toxicity 32. However, there is an evident controversy regarding the miR-200 family in AD. Nevertheless, it is known that miR-200c targets and regulates multiple mRNAs, and its abnormal expression can be used to characterize specific diseases and disease progression stages. Therefore, the specific role of miR-200c in AD and the mechanisms underlying its action are not clearly understood.

In the current study, we investigated the effects of miR-200c abnormal regulation on AD pathology in 5xFAD mice. First, we observed that miR-200c was decreased in the hippocampus of 4-month-old 5xFAD mice among 1247 miRNAs screened through microarray analysis. The discrepancy between our results and previously reported results are attributed to the difference in pathological progression time between AD animal models. In addition, miR-200c increases up to 6 months of APP/PS1 mice, but miR-200c expression decreases at 9 months of APP/PS1 mice, where both Aβ, phosphorylated tau, and cognitive impairment are observed 32. Thus, the expression of miR-200c can change according to the time when the pathological characteristics and cognitive impairment of AD appear. Our results confirmed that miR-200c plays a role in regulating genes in early AD.

Second, we explored AD-related genes associated with miR-200c and predicted that they could be related to 14-3-3γ. 14-3-3 is an adapter protein that is abundant in the brain and is involved in broad-spectrum regulation of both general and specialized signaling pathways through recognition of a phosphoserine or phosphothreonine motif 33, 34. Recently 14-3-3 proteins are known as novel pharmacological targets in neurodegenerative diseases 35. 14-3-3 overexpression reduces the tau binding of microtubules, increasing the tubulin instability 36. Increased 14-3-3 increases tau phosphorylation and aggregation 37, 38. Overexpression of 14-3-3ζ, one of 14-3-3 families, promotes tau phosphorylation and the degradation of synaptophysin 39. This suggests that 14-3-3 is associated with tau phosphorylation, which may be associated with AD pathology. So, we confirmed that miR-200c regulates 14-3-3γ by performing a dual-luciferase assay in HEK293 cells. In addition, the mRNA level of 14-3-3γ did not change; however, the protein level increased when the miR-200c inhibitor was inhibited by transfection in SH-SY5Y and primary hippocampal neurons. This suggests that miR-200c was associated with AD pathology and regulated 14-3-3γ.

Third, a reduction in miR-200c was observed to activate the 14-3-3γ-GSK-3β-p-tau pathway in primary hippocampal neurons. Previously, 14-3-3 proteins were increased in the brains of patients with AD 40, particularly in the neurofibrillary tangles 41. 14-3-3 connects GSK-3β to tau within a brain microtubule-associated tau phosphorylation complex 19. Additionally, GSK-3β phosphorylates the tau protein at multiple sites in intact cells 21. We confirmed that 14-3-3γ and p-GSK-3β were increased in the hippocampus of 4-month-old 5xFAD mice. In addition, p-GSK-3β and p-Tau levels were increased in SH-SY5Y and primary hippocampal neurons transfected with the miR-200 inhibitor. Also, the increased GSK-3β activity through miR-200c inhibition was inhibited by AR-A014418 that GSK-3β selective inhibitor, which confirmed that phosphorylated tau decreased. These suggest that miR-200 inhibition increases p-tau by activating GSK-3β, as it regulates the translation of 14-3-3γ.

Fourth, inhibition of miR-200c affected memory impairment and increased tau phosphorylation in normal mice. Transfection of a miR-200c inhibitor into the hippocampus of C57BL/6 mice resulted in memory impairment and an increase in p-tau, along with the increase in 14-3-3γ levels and GSK-3β activity. These *in vivo* changes were consistent with those observed *in vitro*. Thus, memory deficits induced by the miR-200c inhibitor injected into the hippocampus were associated with the predicted alterations in the 14-3-3γ-p-GSK-3β-p-tau pathway. These results support the hypothesis that miR-200c inhibition increases tau hyperphosphorylation.

miRNA can be secreted from the brain into extracellular space, and secreted miRNAs are detected in the serum, which is related to the disease status, neuropsychological assessment, and pathological characteristics of AD 16, 42, 43. So, we performed RT-qPCR in human blood serum to confirm that miR-200 expression was regulated in human AD. We found significantly decreased relative expression of miR-200c in the serum of AD patients compared to the control group, significant correlations of serum miR-200c levels with MMSE scores, indicating specific dependence on disease progression. The AUC measured for miR-200c was 0.7127, which demonstrates a high sensitivity and specificity for AD. This result suggests that serum miR-200c levels can be independently applied to the auxiliary diagnosis of AD.

Owing to the limitations of this study, the results do not confirm that the AD pathology was alleviated when miR-200c was increased in 5xFAD mice. If additional experiments are performed from the perspective of miR-200c expression with disease progression, we can improve our understanding of AD pathophysiology and possibly determine an optimal time point for the initiation of therapeutic intervention before the occurrence of irreversible neuronal changes. In addition, our clinical study is preliminary, and some limitations of this study should be noted. Additionally, the diagnostic model based on the serum miR-200c level and MMSE score should be validated in a much larger cohort, and more accurate diagnostic models must exist to further explore this diagnostic model.

Despite these restrictions, this study revealed that miRNA changes contribute to AD pathology and can affect memory impairment and tau pathology. Considering that the changes in miR-200c expression occurred alongside behavioral disturbances and miR-200c is decreased in the serum of AD patients, miR-200c downregulated expression may serve as a promising diagnostic biomarker of AD.

## Supplementary Material

Supplementary figures.

## Figures and Tables

**Table 1:** List of primers used in this study

Primer Name	Sequence (5'- 3')
Ywhag-Forward	TTCGGTTTCCTTCTTTCCAGCC
Ywhag-Reverse	TGGTTCATTCAGCTCTGTCACG
GAPDH-Forward	CCCCCAATGTATCCGTTGTGG
GAPDH-Reverse	TCCTCAGTGTAGCCCAGGATG

**Table 2:** List of antibodies used in this study

Antibody	Company	Cat No.	M.W. (kDa)		Source
14-3-3γ	Santa Cruz	SC-731	28		Rabbit
p-GSK-3β (Y216)	Santa Cruz	SC-135053	47		Rabbit
GSK-3β	Santa Cruz	SC-9166	47		Rabbit
AT-8	Thermo Fisher Scientific	MN1020	45-60		Mouse
AT-180	Thermo Fisher Scientific	MN1040	45-60		Mouse
Tau H-150	Santa Cruz	SC-5587	45-60		Rabbit
GAPDH	Bioworld	AP0066	36		Rabbit
β-actin	Santa Cruz	SC-47778	42		Mouse
Goat Anti-Mouse IgG (H + L)-HRP Conjugate	Bio-rad	#170-6516			
Goat Anti-Rabbit IgG (H + L)-HRP Conjugate	Bio-rad	#170-6515			

**Table 3:** DAVID functional annotation

Category	Term	Count	%	EASE	Bonferroni	Benjamini
UP_KEYWORDS	Phosphoprotein	637	63.50947	1.12E-87	3.56E-85	3.56E-85
UP_KEYWORDS	Nucleus	359	35.79262	4.40E-33	1.39E-30	6.97E-31
GOTERM_MF_DIRECT	Protein binding	358	35.69292	7.72E-30	7.38E-27	7.38E-27
UP_KEYWORDS	Alternative splicing	357	35.59322	9.32E-28	2.96E-25	9.85E-26
GOTERM_CC_DIRECT	nucleus	441	43.9681	1.23E-26	7.04E-24	7.04E-24

**Table 4:** Demographic and clinical parameters of studied groups

Variables	Control	AD
Number of subjects	28	22
Female (F)-Male (M)	18 (F) - 10 (M)	15 (F) - 7(M)
Age (years) (mean ± SEM)	69.89 ± 1.10	76.45 ± 1.89
MMSE (mean ± SEM)	27.89 ± 0.28	14.86 ± 0.77****
CDR (mean ± SEM)	0.25 ± 0.05	0.93 ± 0.10 ****
GDS (mean ± SEM)	1.17 ± 0.18	4.27 ± 0.19 ****

AD, dementia in Alzheimer's disease; MMSE, Mini-Mental Status Examination; CDR, Clinical Dementia Rating; GDS, Global Deterioration Scale. **** *p*<0.0001 compared with the control subjects, using student's T-test.

## Abbreviations

3′ UTR: 3′ untranslated regions
Aβ: Amyloid β
AD: Alzheimer's disease
CDR: Clinical Dementia Rating
GDS: Global Deterioration Scale
miRNAs: microRNAs
MMSE: Mini-Mental State Examination
MWM: Morris water maze
NFT: neurofibrillary tangles
p-GSK-3β: GSK-3β phosphorylation
p-tau: Tau phosphorylation
Tg: Transgenic
Thio S: Thioflavin S
WT: Wild-type
