# Supplementary material for: miR-200c suppression increases tau hyperphosphorylation by targeting 14-3-3γ in early stage of 5xFAD mouse model of Alzheimer's disease
Source: Int J Biol Sci. 2022 Mar 6;18(5):2220–34. doi: 10.7150/ijbs.66604 (PMC8935215; doi:10.7150/ijbs.66604)
Supplement: Supplementary file 1 — Supplementary figures. [file ijbsv18p2220s1.pdf]

miR-200c suppression increases tau hyperphosphorylation by targeting 14-3-3γ in early stage of 5xFAD mouse model of Alzheimer's disease

Hyunjun Park[1,2], Yeong-Bae Lee[2,3] and Keun-A Chang[1,2,4,*]

[1]Department of Health Sciences and Technology, Gachon Advanced Institute for Health Sciences and Technology (GAIHST), Gachon University, Incheon, 21999, Korea

[2]Neuroscience Research Institute, Gachon University, Incheon, 21565, Korea

[3]Department of Neurology, Gil Medical Center, College of Medicine, Gachon University, Incheon 21565, Korea

[4]Department of Pharmacology, College of Medicine, Gachon University, Incheon, 21999, Korea

[*]**Correspondence:** keuna705@gachon.ac.kr

Supplementary Figures

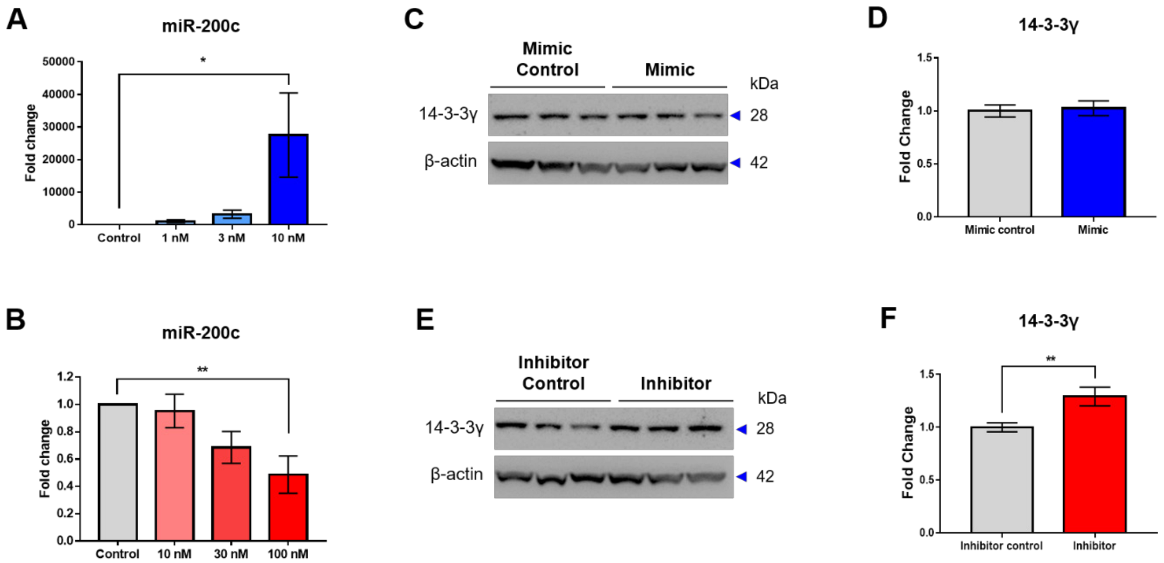

Supplementary figure 1. miR-200c regulates 14-3-3γ expression in SH-SY5Y.

(A-B) After transfection of mimic or inhibitor in SH-SY5Y, expression of miR-200c was evaluated using RT-qPCR (n = 6-8). (C-F) The expression of 14-3-3γ protein in SH-SY5Y after transfection of miR200-C mimic or inhibitor was analyzed by Western blotting. The immunoreactivity of p-GSK-3β/GSK-3β (D&F) was relatively quantified by image J, normalized to β-actin internal control (n = 10-11). Data represent the mean ± SEM. Data were replicated in a minimum of independent three times cultured samples. **p*<0.05 and ***p*<0.01. Data of miR-200c expression were analyzed by one-way ANOVA test, and western blot data were analyzed student's T-test.

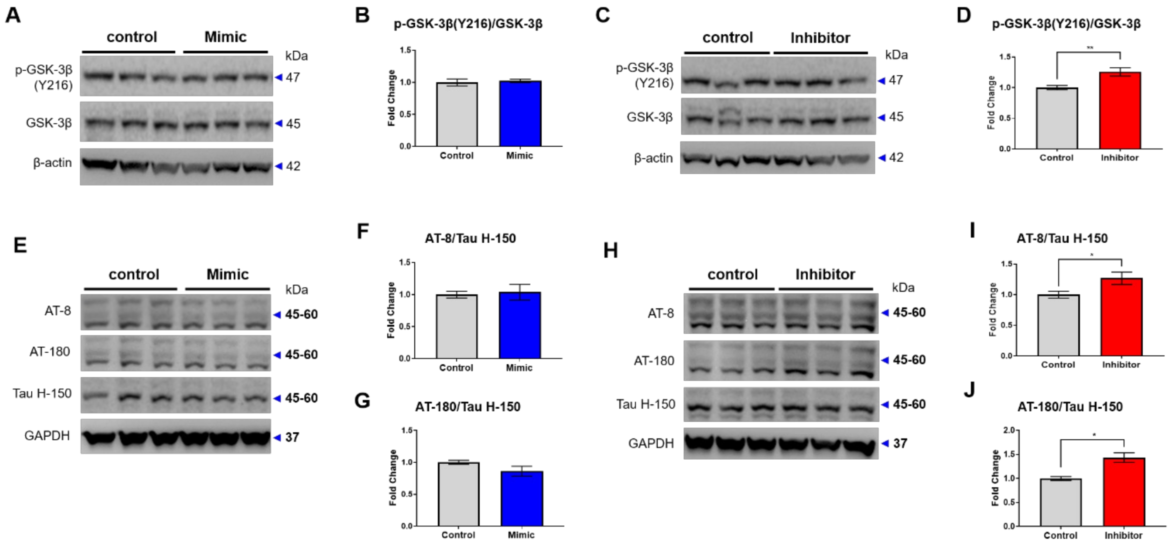

Supplementary figure 2. Changes in expressions of p-GSK-3β and p-tau by miR-200c mimic and inhibitor in SH-SY5Y.

(A-D) The expression of p-GSK-3β (Y216), GSK-3β protein in SH-SY5Y after transfection of miR200-C mimic (A-B) or inhibitor (C-D) were analyzed with Western blotting. The immunoreactivity of p-GSK-3β/GSK-3β (B&D) was relatively quantified by image J, normalized to β-actin internal control. (E-J) Expression of phosphorylated tau proteins (AT-8 and AT-180) and tau protein (Tau H-150) in SH-SY5Y after transfection of miR200-C mimic (E-G) or inhibitor (H-J) were analyzed with Western blotting. The immunoreactivities of AT-8/Tau H-150 (F&I), and AT-180/Tau H-150 (G&J) were relatively quantified by image J, normalized to GAPDH internal control. Data represent the mean ± SEM (n=7-8). Data were replicated in a minimum of independent three times cultured samples. *p<0.05, **p<0.01, and ns = non-significant. All data were analyzed student's T-test.

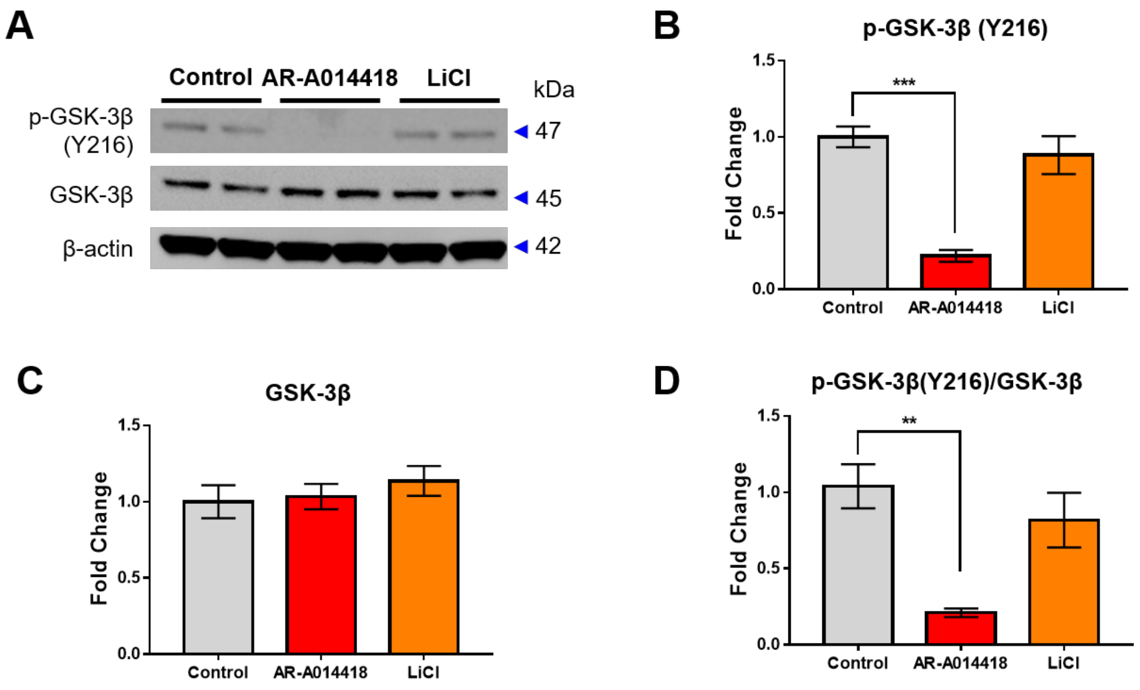

Supplementary figure 3. GSK-3beta inhibitor reduces the GSK-3β phosphorylation.

(A) The expression of p-GSK-3β (Y216), GSK-3β protein in SH-SY5Y after treatment of GSK-3β inhibitor (AR-A014418, LiCl) were analyzed with Western blotting. (B-D) The immunoreactivity of p-GSK-3β (Y216) (B), GSK-3β (C), and p-GSK-3β/GSK-3β (D) were relatively quantified by image J, normalized to β-actin internal control. Data represent the mean ± SEM. Data were replicated in a minimum of independent three times cultured samples. **$p<0.01$, ***$p<0.001$, and ns = non-significant. All data were analyzed one-way ANOVA test.
